# Modeled changes to the Great Plains low‐level jet under a realistic irrigation application

**DOI:** 10.1002/asl.888

**Published:** 2019-02-28

**Authors:** S. Arcand, L. Luo, S. Zhong, L. Pei, X. Bian, J.A. Winkler

**Affiliations:** ^1^ Department of Geography, Environment, and Spatial Sciences Michigan State University East Lansing Michigan; ^2^ Department of Earth and Environmental Sciences Michigan State University East Lansing Michigan; ^3^ United States Forest Service Northern Research Station East Lansing Michigan

**Keywords:** Great Plains, irrigation, low‐level jet

## Abstract

Low‐level jets (LLJs) are relatively fast‐moving streams of air that form in the lower troposphere and are a common phenomenon across the Great Plains (GP) of the United States. LLJs play an important role in moisture transport and the development of nocturnal convection in the spring and summer. Alterations to surface moisture and energy fluxes can influence the planetary boundary layer (PBL) development and thus LLJs. One important anthropogenic process that has been shown to affect the surface energy budget is irrigation. In this study, we investigate the effects of irrigation on LLJ development across the GP by incorporating a dynamic and realistic irrigation scheme into the Weather Research and Forecasting (WRF) model. WRF simulations were conducted with and without the irrigation scheme for the exceptionally dry summer of 2012 over the GP. The results show irrigation‐introduced changes to LLJ features both over and downstream of the most heavily irrigated regions in the GP. There were statistically significant increases to LLJ speeds in the simulation with the irrigation parameterization. Decreases to the mean jet core height on the order of 50 m during the overnight hours were also simulated when irrigation was on. The overall frequency of jet occurrences increased over the irrigated regions by 5–10%; however, these differences were not statistically significant. These changes were weaker than those reported in earlier studies based on simple representations of irrigation that unrealistically saturate the soil columns over large areas over a long period of time, which highlights the importance and necessity to represent human activity more accurately in modeling studies.

## 1 INTRODUCTION

Wind maxima in the lower atmosphere, referred to as low‐level jets (LLJs), are especially frequent over the Great Plains (GP) of the United States (Bonner, 1968), where they contribute to moisture transport and the initiation and maintenance of nocturnal convection (Arritt *et al.*, 1997; Cook *et al.*, 2008). LLJs are also associated with heavy precipitation and severe weather in this region (Means 1954; Arritt *et al.*, 1997). Heat and moisture exchanges within the planetary boundary layer (PBL) play an important role in LLJ development (Shapiro *et al.*, 2016). Alterations to these fluxes, such as those induced by changes in soil moisture from agricultural irrigation (Basara and Crawford, 2002; Harding and Snyder, 2012a; 2012b), can be expected to impact the frequency and strength of LLJs (Adegoke *et al.*, 2003).

Land use across the GP is dominated by agriculture, with over 6 million hectares of irrigated farmland (Maupin *et al.*, 2014). Until now, only a few studies assessed the potential effects of soil moisture, more generally, and irrigation, specifically, on LLJs in the GP. Each of these studies is sensitivity experiments that assumed unrealistic amounts of soil water and/or unrealistic irrigation applications. For example, McCorcle (1988) increased soil moisture without considering land cover type and found that modulation of soil moisture can affect low‐level jet velocities. In McCorcle (1988), as simulated by a boundary‐layer model, the elevation‐related geographical variation in soil saturation across the GP resulted in circulations along boundaries of varying soil moisture that can increase (decrease) jet speeds with the circulation being in (out of) phase with the nocturnal inertial oscillation (Blackadar, 1957). Huber *et al.* (2014), in contrast, took differing land use types into account but applied an unrealistic amount of irrigation water, saturating the entire top meter of the soil daily throughout their model simulation. They found that LLJ speeds decreased under higher soil moisture conditions, which they argued was due to evaporative cooling weakening the regional scale pressure gradients. The potential limitations of these earlier studies are highlighted by the findings of Zhong and Doran (1997), who showed that the simplified assumptions of the amount and/or spatial distribution of soil moisture often used in idealized numerical simulations tend to result in gross overestimations of the response of the lower atmosphere to the underlying heterogeneous surface forcing.

More recent LLJ research conducted with data from the PECAN (Plains Elevated Convection at Night) (Geerts *et al.*, 2017) field campaign has found new insights into the effects of sloped terrain and the relative contributions of Blackadar's (Blackadar, 1957) inertial oscillation theory and Holton's (Holton, 1967) differential heating mechanisms of LLJ development. Parish (2017) found that on seasonal scales the continued differential heating over sloped terrain resulted in thermal wind contributions from layers above the main LLJ level strengthened the background geostrophic wind aiding in LLJ development. Fedorovich *et al.* (2017) conducted direct numerical simulations of idealized LLJ cases to investigate the role of gently sloping terrain and turbulent kinetic energy on LLJ formation and intensity. LLJ profiles and wind maxima were shown to be sensitive to the slope angle and the magnitude of the surface buoyancy. They found LLJ intensity increases associated with an increase in surface buoyancy prior to sunset.

Given the limitations of earlier studies as well as more recent research contributions, the goal of this research is to better understand potential changes in the characteristics of LLJs in the GP under a realistic representation of irrigation and the respective changes in soil moisture. We employ numerical simulations for the summer of 2012 to address the research question of the effects of irrigation on the GP LLJ. This study period was chosen due to the relatively hot and dry conditions over the GP where irrigation strongly exceeds precipitation and provides optimal irrigation signals on the seasonal scale.

## 2 METHODS

### 2.1 Model setup

Numerical simulations were conducted using the Weather Research and Forecasting (WRF) model (v3.6) over the entire conterminous United States to account for both local and downstream effects of irrigation on the seasonal scale. A dynamic irrigation scheme was earlier incorporated into this version of WRF by Pei *et al.* (2016). The model simulations were configured on a single mesh with 30‐km horizontal grid spacing, 30 vertical layers, and driven by the North American Regional Reanalysis (NARR; Mesinger *et al.*, 2006). The Kain–Fritsch cumulus parameterization scheme (Kain and Fritsch, 1993; Kain, 2004) was used for deep moist convection. The WSM6 6‐class graupel (Hong and Lim, 2006) and the YSU schemes (Hong *et al.*, 2006) were used for microphysics and PBL processes, respectively. Shortwave and longwave terrestrial radiation processes were parameterized with the Dudhia shortwave (Dudhia, 1989) and the RRTM longwave radiation (Mlawer *et al.*, 1997) schemes. As the PBL process and the cumulus parameterizations are critical to represent the LLJ and rainfall characteristics, this set of physics schemes were deliberately selected to reflect the least precipitation bias in the control run (without irrigation) based on four sets of parameterization sensitivity tests.

The WRF model characterizes the land surface using the United States Geological Survey's (USGS) 24 land‐use/land‐cover categories. The Noah‐Mosaic module (Li *et al.*, 2013) was chosen to obtain subgrid‐scale information on land use/land cover type. The eight dominant subgrid land use/land‐cover types within each 30‐km WRF grid cell were used to capture land use heterogeneity, and the “irrigated cropland and pasture” category is irrigated at the subgrid scale during the simulations.

### 2.2 Irrigation scheme

**Figure 1:**
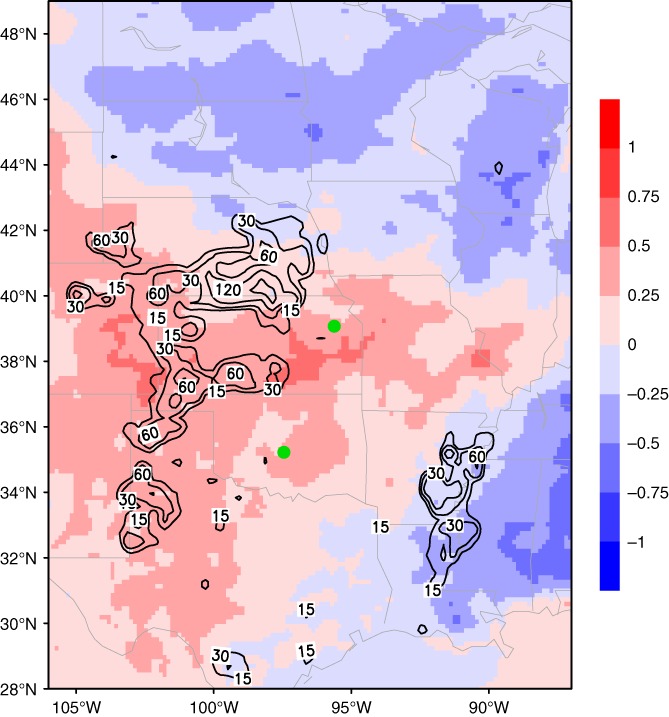
Total amount of added irrigation water (kg/m^2^) during the 3‐month study period (black contours) and the mean difference (IRR‐NOIR) in surface temperature (color shading, unit: °C) between the two simulations. The green dots indicate rawinsonde station locations used for validation

Irrigation in the GP was predominantly applied via sprinkler systems and was included in the WRF simulations as added precipitation. The irrigation trigger depended on the plant available water within Noah–Mosaic's second soil layer (10–40 cm below ground), and once triggered, water was applied for 2 hr at 20 mm/hr within only the designated fraction of each grid cell. Figure 1 shows the total amount of simulated irrigation for the three‐month study period and was shown to be a decent representation when compared with the USGS water‐use report (Maupin *et al.*, 2014) by Pei *et al.* (2016). A more detailed description of the irrigation scheme and its validation can be found in Pei *et al.* (2016).

### 2.3 Experimental setup and jet identification

Two simulations were run, one with the addition of the irrigation scheme (IRR) and the other without the irrigation scheme (NOIR). The two simulations ran from June through August (JJA), 2012, with a one‐month spin up. LLJs in the model simulations were identified using a modified version of Bonner's (Bonner, 1968) original criteria that was previously used by Doubler *et al.* (2015). Vertical wind profiles qualified as a LLJ if the maximum wind speed was at least 12 m/s; if the wind speed decreased by at least 6 m/s from the level of the jet core to the next minimum or to 5‐km above ground level (AGL), whichever was lower; and if the wind speed decreased by at least 6 m/s between the level of the jet core and the surface.

## 3 RESULTS

### 3.1 WRF model validation

To evaluate the ability of the WRF simulations to reproduce the climatological features of the LLJ, we first compared the spatial patterns of average jet speed, elevation, and frequency obtained from the IRR simulation to those obtained directly from NARR wind fields for the study period. NARR was selected over rawinsonde observations for this validation because of its finer spatial and temporal resolution compared to the twice‐daily observations from the coarse rawinsonde network. Moreover, Walters *et al.* (2014) earlier compared LLJ characteristics obtained from rawinsondes to those obtained from nearby NARR grid points and found that, although NARR tends to underestimate LLJ frequency compared to rawinsonde observations, NARR is nonetheless a viable proxy for representing the climatological characteristics of LLJs. In addition, Doubler *et al.* (2015) showed that the NARR wind fields capture the long‐term climatological characteristics of LLJs in North America, including jets in the GP.

**Figure 2:**
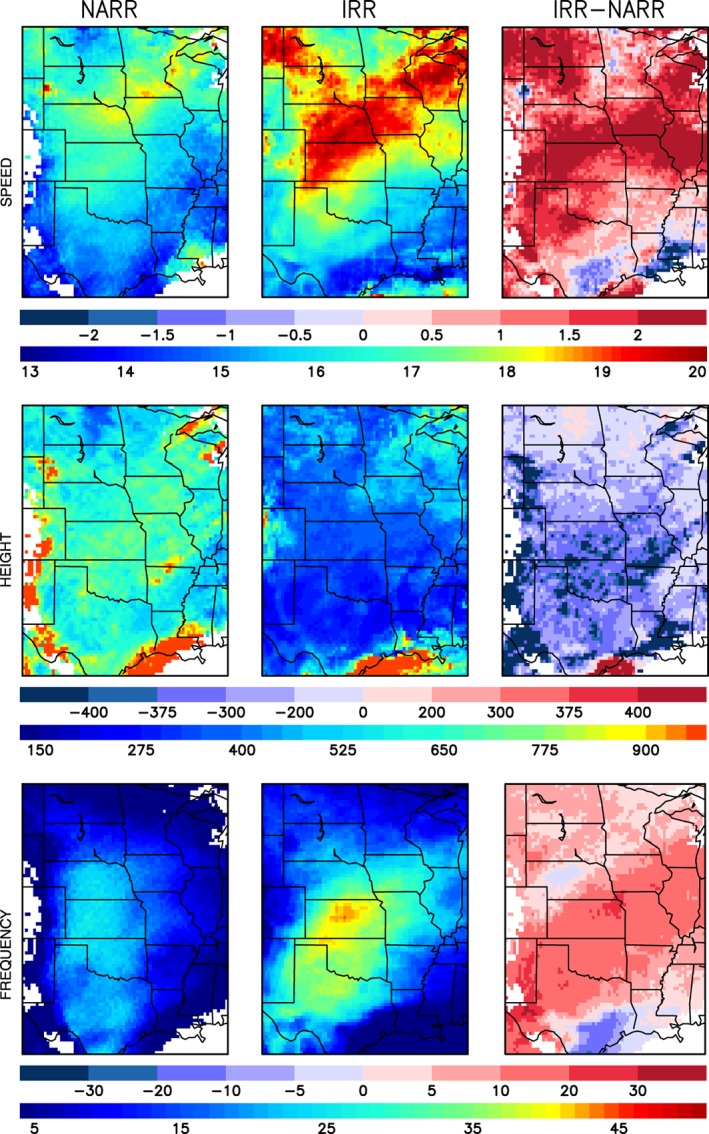
Differences between the IRR simulation and NARR in LLJ mean speed (m/s) and height (m) and jet frequency (% of all time steps with LLJ profiles)

The WRF‐simulated jet speeds for the study period compare reasonably well to the NARR speeds although some differences are evident (Figure 2). The simulated jet speeds are larger, with maximum wind speeds over 20 m/s compared to maximum speeds of 16–17 m/s for NARR. The IRR simulation also expands the spatial extent of maximum winds. A broad area of intense jet speeds stretches from the Texas panhandle northward to the U.S.–Canadian border for the IRR simulation, whereas the strongest winds are confined to a smaller area from northeast Nebraska and southern Minnesota in NARR. The simulated jet core heights are on average about 300 m lower (i.e., closer to the surface) than the average heights from NARR. Also, the simulated jet elevations are fairly uniform across the GP, varying between 300 and 500 m AGL, whereas the NARR jet elevations are mostly above 600 m AGL. Compared to NARR, the IRR simulation overestimates jet frequency by approximately 10–20%. Jet frequency is defined here as the number of 6‐hourly time steps during the study period when the LLJ criteria were met. In addition, the area of maximum jet occurrence in the IRR simulation is oriented southwest‐to‐northeast from west‐central Texas to Iowa rather than oriented from south‐to‐north as seen for NARR.

**Figure 3:**
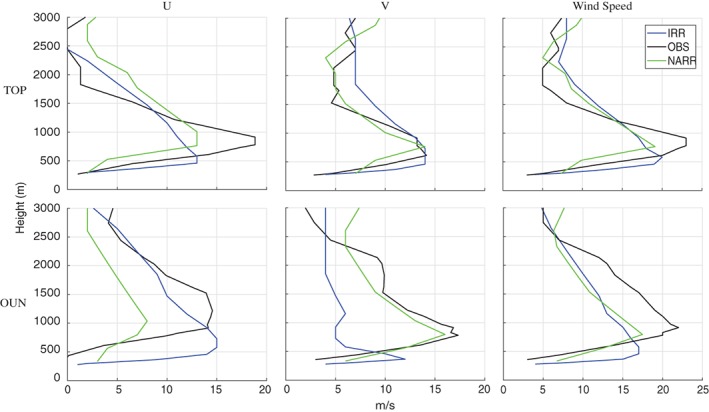
U, V, and wind speed (1200 UTC) profiles Topeka, KS (TOP) on July 4, 2012, and Norman, OK (OUN) on July 25, 2012

Rawinsonde observations, with their finer vertical resolution, were used in addition to NARR to evaluate how well the IRR simulation captures the observed vertical wind profiles during LLJ events. Rawinsonde observations from 1200 UTC July 4 at Topeka, KS, and from 1200 UTC July 25, 2012 at Norman, OK, were compared to vertical profiles obtained from the nearest IRR and NARR grid points. LLJ maximum wind speed and height are greater in the rawinsonde observations than either the simulated or NARR wind profiles (Figure 3).

In sum, the WRF IRR simulation captured the overall characteristics of LLJs during the study period. Differences with observations are relatively modest, with the LLJs in the IRR simulation slightly stronger and more frequent compared to LLJs identified from NARR and are located at lower elevations compared to jets seen in rawinsonde observations.

### 3.2 IRR and NOIR comparison

**Figure 4:**
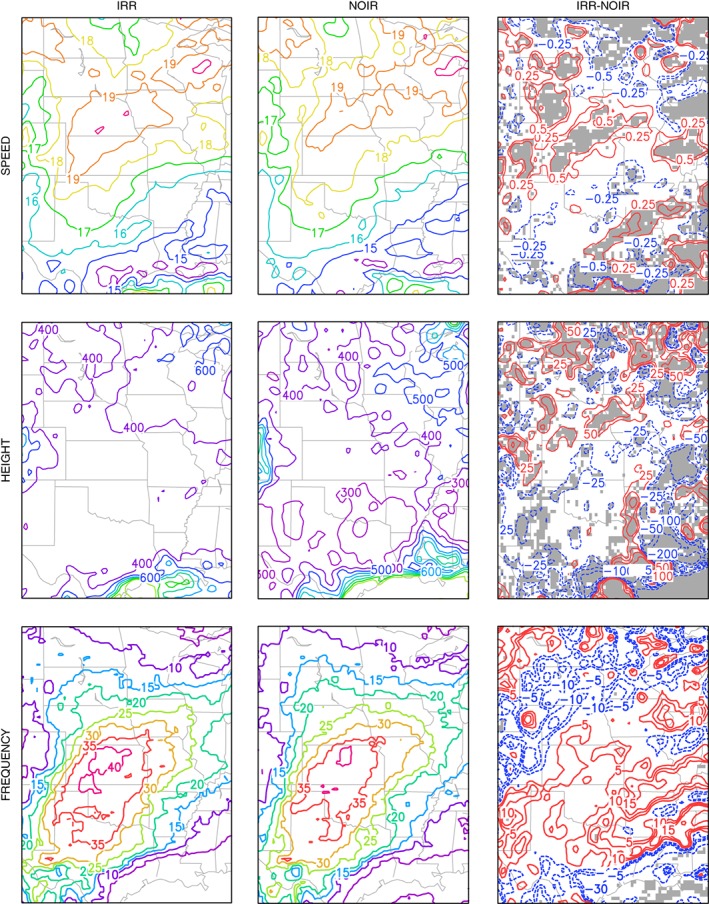
JJA mean and difference of LLJ core speed (m/s) and height (m), and frequency (%) for the IRR and NOIR simulations. Shaded areas represent grid cells where the difference is statistically significant

Differences between the IRR and NOIR simulations (IRR‐NOIR) are observed for several jet characteristics (Figure 4). A statistically significant (95% confidence level) increase in JJA (i.e., seasonal) mean jet speed of approximately 1 m/s is found over portions of Kansas and Nebraska where the majority of irrigation water was applied for the IRR simulation (Figure 1). This is also an area of increased jet frequency, with 5% more 6‐hourly vertical wind profiles meeting the LLJ criteria for the IRR simulation compared to the NOIR simulation. In contrast, mean jet speed decreased by approximately 1 m/s in northern Minnesota, southern Wisconsin/northern Indiana, and southeast Texas/southern Louisiana, although these differences should be interpreted cautiously as jet frequency is small in these areas. Decreases in jet frequency in the IRR simulation are also seen for these areas. On the other hand, the differences between the two simulations in the height of the level of maximum wind are either statistically insignificant or the spatial patterns of the significant differences are not coherent.

The vertical and diurnal extent of the southerly wind speed differences between the IRR and NOIR simulation is important when addressing changes to the LLJ. The longitudinal cross sections for 39°N latitude shown in Figure S1, Supporting Information display IRR‐NOIR differences in the JJA mean v‐wind component at four time steps (0000, 0600, 1200, 1800 UTC). Evident from the cross sections are the stronger southerly winds below 800 hPa in the IRR simulation at 0600 UTC and 1200 UTC. The largest differences are on the order of 1 m/s and are located at approximately 101°W (western Kansas) at 0600 UTC and expand eastward to 96°W at 1200 UTC.

**Figure 5:**
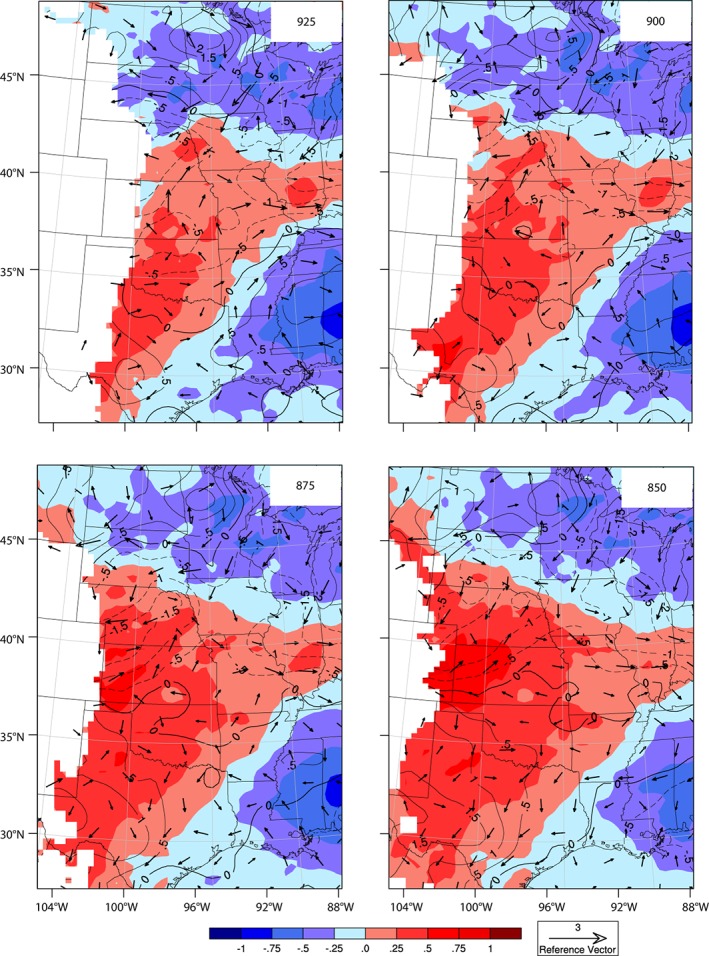
JJA mean 1200 UTC temperature (°C) difference and vector wind (m/s) difference (IRR‐NOIR). Increases in geopotential height (solid black lines) and decreases in geopotential height (dashed black lines) are plotted in m for each pressure level

To evaluate possible reasons for the changes in jet strength and frequency, we considered changes in geopotential height, temperature, and airflow at several pressure levels (925, 900, 875, 850 hPa) at 1200 UTC (Figure 5). The Bermuda High appears to extend farther west in the IRR simulation, and the stronger geopotential height gradient over central and western Kansas and Nebraska may contribute to the stronger wind speeds (Zhu and Liang, 2012) over the areas where more water was applied. In addition, 1200 UTC air temperatures are warmer over the central and southern GP in the IRR simulation compared to the NOIR simulation (night‐time warming due to irrigation), which would act to enhance the east–west air temperature gradient and increase the contribution of the thermal wind to jet formation (Holton, 1967). The generally southerly wind vector differences over Kansas and eastern Nebraska are consistent with this mechanism.

**Figure 6:**
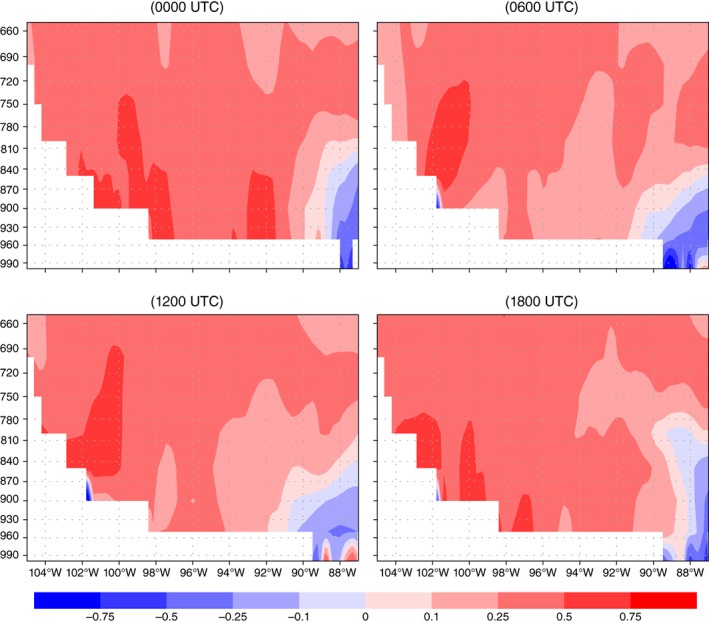
Longitudinal cross section of the JJA mean temperature (°C) differences (IRR‐NOIR) at 37.5°N latitude. The diurnal cycle shown is averaged for four model times, and a clear positive difference can be seen during all times and especially near the surface during the afternoon (1800 UTC) and evening (0000 UTC) hours

The vertical cross section of IRR‐NOIR temperature (Figure 6) shows the depth and extent of the warming across the sloped GP terrain. This warming was seen as one of the primary and important differences after the addition of irrigation. The higher temperatures of just under 1 °C near the surface from about 102°–97°W at 0000 UTC may be interpreted as an increase to near surface buoyancy which Fedorovich *et al.* (2017) correlated to an increase of intensity to subsequent LLJs. This warming, together with the height differences and thermal wind contributions noted above, may together provide enough forcing to help explain the differences between the IRR and NOIR simulations.

## 4 DISCUSSION

In agreement with other studies (Cook *et al.*, 2015), the increase in low level moisture provided by a realistic amount of irrigation is enough to change synoptic‐scale circulation patterns. In the simulated exceptionally dry summer of 2012, as local convection over the GP has already been prohibited by the synoptic conditions, the added irrigation water is mostly advected downstream instead of being recycled as local precipitation (Pei *et al.*, 2016). This suppressed GP local precipitation is believed to be the responsible climatic feedback leading to uncharacteristic warming throughout the irrigated region, since irrigation is generally considered to provide a cooling effect. Figure 1 shows about a 1 °C warming in irrigated areas of the GP and cooling downstream corresponding to the changes in the precipitation pattern.

The results of this study highlight the significance of employing realistic irrigation applications and soil moisture content in numerical simulations of LLJs in the GP. Contrary to prior sensitivity studies conducted over a relative dry May–June–July period for the GP (e.g., Huber *et al.*, 2014), the average speed of the LLJ increased, rather than decreased, when a dynamic and more realistic irrigation scheme was included in numerical simulations. Furthermore, the frequency of LLJs increased. The increase in LLJ speed over the area with largest added irrigation water is consistent with a simulated increase in low‐level temperature during the overnight and early morning hours. This temperature increase may enhance the thermal wind contribution to LLJ formation and strength. It is also possible that the irrigation‐introduced increase to the central US high pressure system noted in Pei *et al.* (2016) may at least be partially responsible for the strengthening of the LLJ.

At the 850‐hPa level Huber *et al.* (2014) found height changes on the order of 6 m, this study found differences on the order of 1 m. In our realistic irrigation simulation and their sensitivity study temperatures changed by about 1 and 4.7 °C, respectively. In this case the sign of the temperature difference is opposite, and the reasons remain unclear as it could be rooted in a range of factors from model configuration to physics parameterizations. Downstream, both studies noted increases to precipitation. Huber *et al.* (2014) found decreases to the LLJ speed on the order of 20–30% between the irrigated and non‐irrigated simulations, or about 2–3 m/s. Also, the magnitude of these changes is on par with other studies which have looked at changes to the LLJ from a climatological perspective with forcing's of similar magnitudes.

McCorcle (1988) investigated the differences in heterogeneous and homogeneous distributions of changing soil moisture and their impact on LLJs. His findings pointed to the importance of analyzing varying spatial distributions of soil moisture. This study acknowledges and elaborates upon this point through the addition of the dynamic and realistic irrigation scheme. By including the Noah‐Mosaic land surface model and differentiating subgrid‐scale land‐use types this model is able to more appropriately handle a realistic and heterogeneous distribution of soil moisture.

Another important aspect of this study is that the simulations were run for a particularly hot and dry year. This was rationalized to maximize the irrigation signal that a realistic irrigation scheme may produce. To expand on this study and the potential feedbacks that could arise due to differing climatological conditions, future work should involve a similar approach but include climatologically normal and wet years. The feedback between irrigation and lower atmospheric circulations may be dependent on the background climate conditions; this may lead to varying responses by LLJs. Also, the parameters used in the irrigation scheme are tuned using data from the GP, and the analyses are thus focused on the GP where the addition of irrigation is most accurate (Pei *et al.*, 2016).

## 5 CONCLUSION

The results of this study show that the LLJ simulation is sensitive to the addition of a realistic irrigation scheme. At the surface, the IRR simulation shows changes in temperature and precipitation. Warming is present through the lower atmosphere over the central US. Increased geopotential heights over the central US and decreased geopotential heights to the north and east resulted in the general changes in precipitation as noted by Pei *et al.* (2016). The changes to jet velocity in this study are slightly reduced in magnitude, and opposite in sign compared to those reported in Huber *et al.* (2014) largely due to the changes to temperature. This study also built upon the spatial distribution issues that were simplified in McCorcle (1988). As expected, results suggest more suppressed effects with reduced amounts of irrigation given the relative difference between this realistic irrigation approach and previous sensitivity tests. However, these differences are still comparable in magnitude over the irrigated areas.

The focus of this study was on the differences in the LLJ due to the addition of a realistic amount and distribution of irrigation. Understanding realistic anthropogenic impacts on the LLJ is critical for the formation of next‐generation atmospheric models. It has been shown that even a relatively modest amount of added irrigation water provides a strong forcing to alter regional to synoptic scale circulations and influence the LLJ. More notably a statistically significant increase of just under 1 m/s in mean LLJ core speeds was found over the irrigated region during the overnight peak jet hours (Figure S1) in the simulated exceptionally dry summer. Acknowledging irrigation's ability to act on the background synoptic forcing should be more carefully considered when developing parameterizations for future climate projections. The difference between this study and earlier sensitivity study also points to the need for more explicit representation of irrigation in operational forecast models.

## Supporting information


**Figure S1.** Longitudinal cross section of the JJA mean *v* wind‐component (m/s) at a latitude of 39°N. The diurnal cycle shown is averaged for four model times, and a clear positive difference can be seen overnight during the peak LLJ times showing an increase in the southerly component of the wind in the IRR simulation.
