# Supplementary material for: Modeled changes to the Great Plains low‐level jet under a realistic irrigation application
Source: Atmos Sci Lett. 2019 Feb 28;20(3):e888. doi: 10.1002/asl.888 (PMC6555437; doi:10.1002/asl.888)
Supplement: Supplementary file 1 — Figure S1. Longitudinal cross section of the JJA mean v wind‐component (m/s) at a latitude of 39°N. The diurnal cycle shown is averaged for four model times, and a clear positive difference can be seen overnight during the peak LLJ times showing an increase in the southerly component of the wind in the IRR simulation. [file ASL2-20-na-s001.docx]

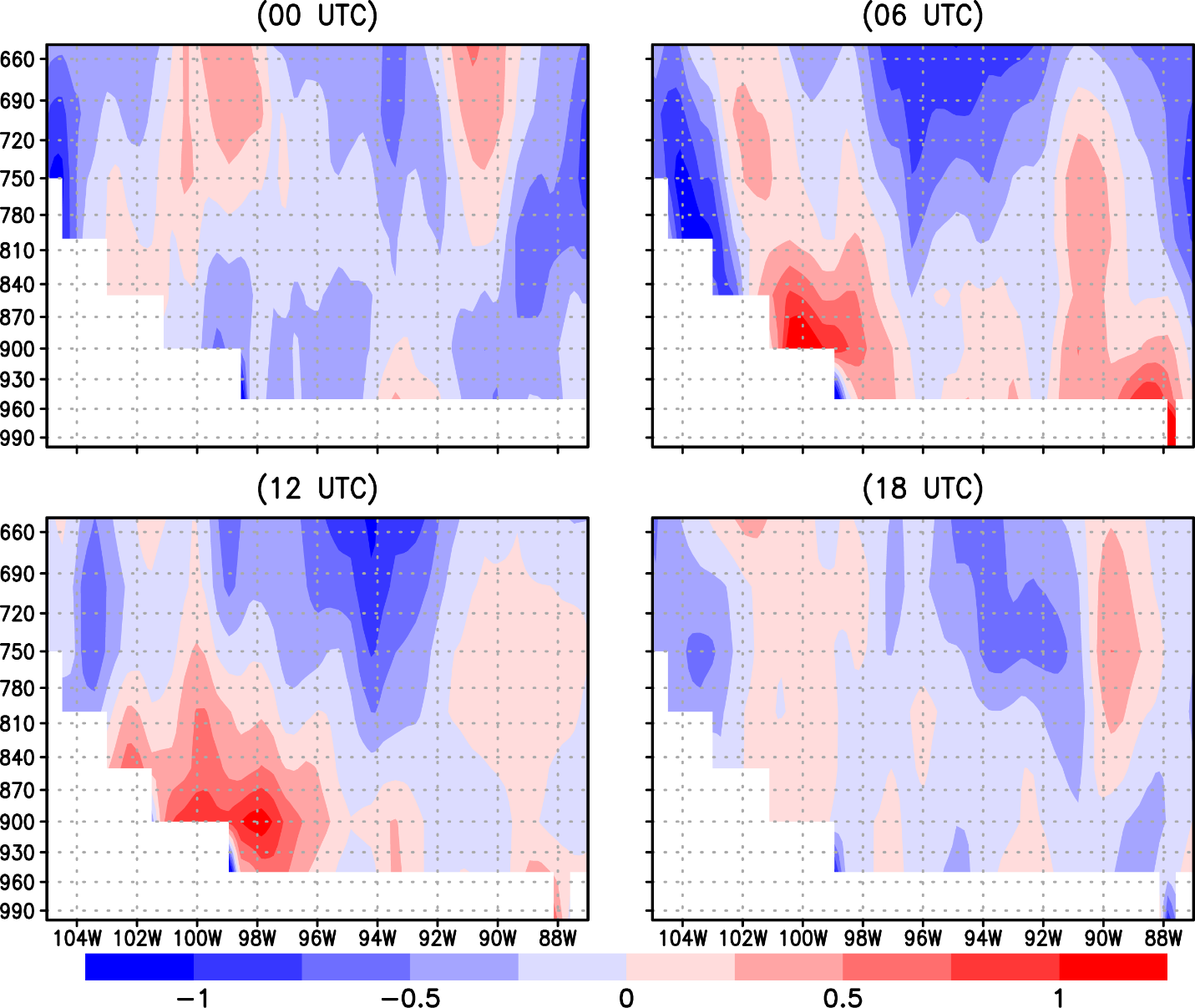


Figure S1. Longitudinal cross-section of the JJA mean v wind-component (m s^-1^) at a latitude of 39° N. The diurnal cycle shown is averaged for four model times, and a clear positive difference can be seen overnight during the peak LLJ times showing an increase in the southerly component of the wind in the IRR simulation.
